# Transcriptional differences between major *Fusarium* pathogens of maize, *Fusarium verticillioides* and *Fusarium graminearum* with different optimum growth temperatures

**DOI:** 10.3389/fmicb.2022.1030523

**Published:** 2022-12-01

**Authors:** Ning Liu, Yue Chen, Jing Liu, Qianfu Su, Bin Zhao, Manli Sun, Hui Jia, Zhiyan Cao, Jingao Dong

**Affiliations:** ^1^State Key Laboratory of North China Crop Improvement and Regulation, Baoding, China; ^2^Hebei Key Laboratory of Plant Physiology and Molecular Pathology, Baoding, China; ^3^Plant Pathogenic Mycotoxin and Molecular Plant Pathology Laboratory, Hebei Agricultural University, Baoding, China; ^4^Jilin Academy of Agricultural Sciences, Changchun, China

**Keywords:** *Fusarium verticillioides*, *Fusarium graminearum*, optimum temperature, transcriptome analysis, thermal ecological niches

## Abstract

*Fusarium verticillioides* and *Fusarium graminearum* are important pathogens causing disease in maize (*Zea mays*) worldwide. The distributions of these fungal pathogens vary greatly in different regions and in different years, and are influenced by environmental and climatic conditions. Temperature has significant effects on the growth and mycotoxin production of *Fusarium* species. In this study, the effects of temperature on the growth and pathogenicity of *F. verticillioides* and *F. graminearum* were investigated. *F. verticillioides* grew fastest and exhibited the strongest pathogenicity to maize stems and grains at 30°C, while *F. graminearum* grew best at 20°C. Both species produced more toxins at 20°C than at 30°C. To explain the interspecific differences in the relationship of growth and temperature, RNA-seq was used to compare *F. verticillioides* and *F. graminearum* cultivated for 4 d at the optimum temperatures of 30°C and 20°C, respectively. Samples of *F. verticillioides* were also cultivated for 9 d (to maximize toxin production) at 20°C and 30°C and analyzed by RNA-seq to investigate the influence of temperature for different growth stages. The differently expressed genes (DEGs) were identified by comparison of cultures grown for the same amount of time but at different temperatures. GO enrichment analysis showed high enrichment of DEGs in categories of membrane part, catalytic activity, metabolic process, and growth at warmer temperature resulted in more down-regulated DEGs enriched in membrane components in all groups. KEGG analysis revealed enrichment of DEGs related to different temperatures in carbohydrate and amino acid metabolism pathways. For both species, there was decreased expression of many DEGs related to amino acid metabolism when cultivated at warm temperature, such as genes related to beta-alanine metabolism and arginine and proline metabolism. However, changes in genes related to glyoxylate and dicarboxylate metabolism and fatty acid degradation were more related to the growth state. The results showing different responses pattern of these pathways provides a foundation for further investigation of the molecular mechanisms underlying distinct thermal ecological niches of *F. verticillioides* and *F. graminearum*.

## Introduction

*Fusarium* species are major *Fusarium* pathogens of maize, causing maize root rot, sheath rot, stalk rot, and ear rot all over the world (Gai et al., 2018; Li et al., 2019). Stalk rot caused by *Fusarium* species leads to yield loss due to lodging and poor grain filling, and mycotoxin contamination of maize kernels caused by *Fusarium* infection directly threatens the health of humans and livestock (Mukanga et al., 2010; Yu et al., 2016). *Fusarium* accumulated in soil and plant residues in fields serve as an important primary source of infection, and when weather and other environmental conditions are suitable, the disease outbreaks occur (Paugh et al., 2021). For example, stalk rot outbreaks are more prevalent in hot and rainy summer conditions (Shi et al., 2017). Many *Fusarium* species can infect maize, mainly including *F. verticillioides*, *F. graminearum*, *F. oxysporum*, and *F. equiseti*, and maize can be infected by a single species or by communities of mixed species (Volker et al., 2013; Qin et al., 2014). However, the distributions of the *Fusarium* species that cause maize rot vary significantly across field locations and years, which is greatly affected by environmental conditions including temperature (Mei, 2003; Fu et al., 2015).

Among the various abiotic factors related to environmental climate, temperature is one of the most critical factors for organisms. Temperature regulates the growth of fungi and also affects the pathogenicity of plant pathogenic fungi (Ezrari et al., 2021). Higher temperature promotes systemic infection of maize by *Fusarium verticillioides* (Murillo-Williams and Munkvold, 2008), and disease severity of wheat *Fusarium* crown rot increases with increasing temperature (Poole et al., 2013). The mechanisms of fungi response to high or low temperature stresses have been examined extensively. Fungi have evolved complex molecular mechanism and regulatory circuits to respond to stress induced by sustained or transient responses to elevated temperature, such as the heat shock response which is one of the most evolutionarily conserved stress responses (Brown et al., 2017; Gomez-Pastor et al., 2018). In addition to directly driving biological activities, temperature can influence intraspecific and interspecific interactions and limits geographic distribution (Fitt et al., 2006). Niche differences can result from differences in response to temperature, leading to separation in space or time. Compared to temperature stress adaptability, fewer studies have investigated the mechanistic basis for optimum temperature selection of different species and how this can affect the niche separation of related species.

In this study we focused on the mechanism of different temperature selection to *F. verticillioides* and *F. graminearum*, which are the two main pathogens of maize ear rot and stalk rot in many areas of the world (Duan et al., 2016; Czembor et al., 2019). *Fusarium verticillioides* and *F. graminearum* produce fumonisins and deoxynivalenol (DON), respectively, the most common mycotoxins (Quesada-Ocampo et al., 2016; Gai et al., 2018). Temperature and water activity have important effects on the growth and mycotoxin production of *Fusarium* species (Peter Mshelia et al., 2020). It is reported that the optimum temperature for growth of *F. verticillioides* is 30°C, while cool growing conditions promote the pathogenesis of *F. graminearum* to maize (Samapundo et al., 2005; Hay et al., 2021). In this study, we investigated the growth rate, conidial production, and pathogenicity to maize stalk and kernels of *F. verticillioides* and *F. graminearum* under different temperatures. The results show that the two species prefer different optimum temperatures for growing and infection. RNA-seq was used to analyze differently expressed genes when grown at optimum temperature. The results of this work will facilitate the prevention of *Fusarium* diseases and the understanding of interspecific ecological differences in temperature selection.

## Materials and methods

### Plant and fungal materials

The maize inbred line B73 and the fungi strains *Fusarium verticillioides* 7600 (Fv) and *Fusarium graminearum* PH-1 (Fg), were obtained from the Key Laboratory of Hebei Province for Plant Physiology and Molecular Pathology.

### Radial growth rate, conidial production, and germination

*Fusarium verticillioides* and Fg were cultivated on potato dextrose agar (PDA) medium at 15, 20, 25, 30, and 35°C, and colony diameter was measured daily for 5 days. The maximum radial growth rate was calculated by plotting and fitting colony growth data. To analyze the influence of cell membrane fluidity on the growth of Fv and Fg, 600 mM dimethylsulfoxide (DMSO) was added in PDA medium to inhibit cell membrane fluidity, and 10 mM benzyl alcohol (BA) was added in PDA medium to increase cell membrane fluidity. To analyze the influence of MAPK signaling pathway on the growth of Fv and Fg, 1 or 3 μM MAKP signaling pathway inhibitor U0126 was added in PDA medium. Fv and Fg incubated at 20 and 30°C to calculate the inhibition rate respectively, and Fv and Fg incubated in PDA medium were used as controls.

*Fusarium verticillioides* and Fg were cultivated in liquid Carboxymethylcellulose medium, with shaking at 180 r/min, at temperatures of 15, 20, 25, 30, and 35°C. After 4 days, the conidia were counted. Conidial suspensions were prepared at 10^6^/ml and applied to water agar plates and cultivated at 20 and 30°C, with microscopic observation every 3 h to calculate the germination rate.

### Pathogenicity assays

Maize was grown in the greenhouse in Baoding (Hebei Agricultural University, Hebei Province, China) maintained at 26°C with a photoperiod of 16 h. Ten-leaf seedlings were used to assay Fv and Fg pathogenicity to maize stems and kernels at 20°C or 30°C, following the methods of Shu et al. (2017) and Xu et al. (2018). The experiment was repeated twice with three replicates for each treatment. Sterile water was used as a control in all assays. The samples were photographed after 4 days. The areas of lesions in the infected stems were calculated and the fungal biomass in the infected kernels was detected using qPCR following the methods previously reported (Saravanakumar et al., 2017).

### Analyses of mycotoxin fumonisins and DON

*Fusarium verticillioides* and Fg were cultivated in potato dextrose medium at 20 and 30°C for 9 days, respectively. The filtered mycelia samples were freeze-dried and weighed and the filtered supernatant was used to extract and detect the mycotoxin fumonisins (FB1 and FB2) and DON following the methods of Li et al. (2019).

### Sample preparation and RNA extraction

The maximum growth rates of two species both occurred at about 4 days of cultivation at their optimum growing temperatures (Supplementary Figure 1). The mycelia of Fv and Fg cultivated on PDA at 20 and 30°C for 4 days, respectively, were collected for transcriptome analysis (samples designated FV4D-20, FV4D-30, FG4D-20 and FG4D-30). The maximum production of fumonisins occurred at 9 days of cultivation of Fv at 20°C (Supplementary Figure 2), so the mycelia of Fv cultivated for 9 days on PDA at 20 and 30°C were also collected for transcriptome analysis (samples named FV9D-20 and FV9D-30).

Mycelia samples were collected by filtration and frozen immediately using liquid nitrogen. Total RNA was extracted with TRIzol reagent and genomic DNA was removed by DNase I treatment (TaKara, Japan). The integrity of the extracted RNA was determined using a 2,100 Bioanalyzer (Agilent) and the concentrations were quantified using a Nanodrop ND-2000 spectrophotometer (Nanodrop Technologies, Wilmington, DE, United States).

### Transcriptome analysis

Sequencing libraries were generated using Illumina Truseq^™^ RNA sample prep Kit (Illumina, San Diego, CA, United States) and then sequenced on Illumina Novaseq 6000 platform.Gene function was annotated based on the following databases: NR (NCBI nonredundant database), Pfam (Protein family), COG (Clusters of Orthologous Groups), Swiss-Prot (a manually annotated and reviewed protein sequence database), Kyoto Encyclopedia of Genes and Genomes (KEGG), and GO (Gene Ontology). The expression level of each gene was calculated using FPKM (fragments per kilobase per million mapped fragments).

Differentially expressed genes (DEGs) were selected based on a log^2^-fold change of 2 and a value of *p* of 0.05. Using this criterium, the numbers of DEGs in there groups are indicated, including the group FV4D by comparing samples FV4D-20 with samples of FV4D-30, the group FG4D by comparing samples FG4D-20 with samples of FG4D-30, and the group FV9D by comparing samples FV9D-20 with samples of FV9D-30. Log2 TPM values were used to produce heatmaps of gene expression. GO enrichment analysis of DEGs was performed by the GOseq R package based on a Kolmogorov–Smirnov test, and the statistical enrichment of DEGs in the KEGG pathway was determined by KOBAS software. BLASTP was used to determine the orthologous genes and characterize gene families.

### qRT-PCR validation

Five genes of Fv and 6 genes of Fg involved in the pathways of analyzed DEGs were used to validate the RNA-seq results through quantitative real-time PCR (qRT-PCR), and the data were processed with the 2^−^ΔΔCt analysis method. Sequences of primers used in this study was listed in Supplementary Table S1.

### Statistical analysis

All analyses were performed using data from three replicates (n = 3). Measurements were then averaged and presented as mean ± standard error (SE). Two-way analysis of variance (ANOVA) and Tukey’s tests using SPSS software (Version 26.0, SPSS Inc., Chicago, IL, United States) evaluated significant differences across all parameters. A value of *p* < 0.05 was accepted as a significant difference.

## Results

### The effect of temperature on growth and conidial production

**Figure 1:**
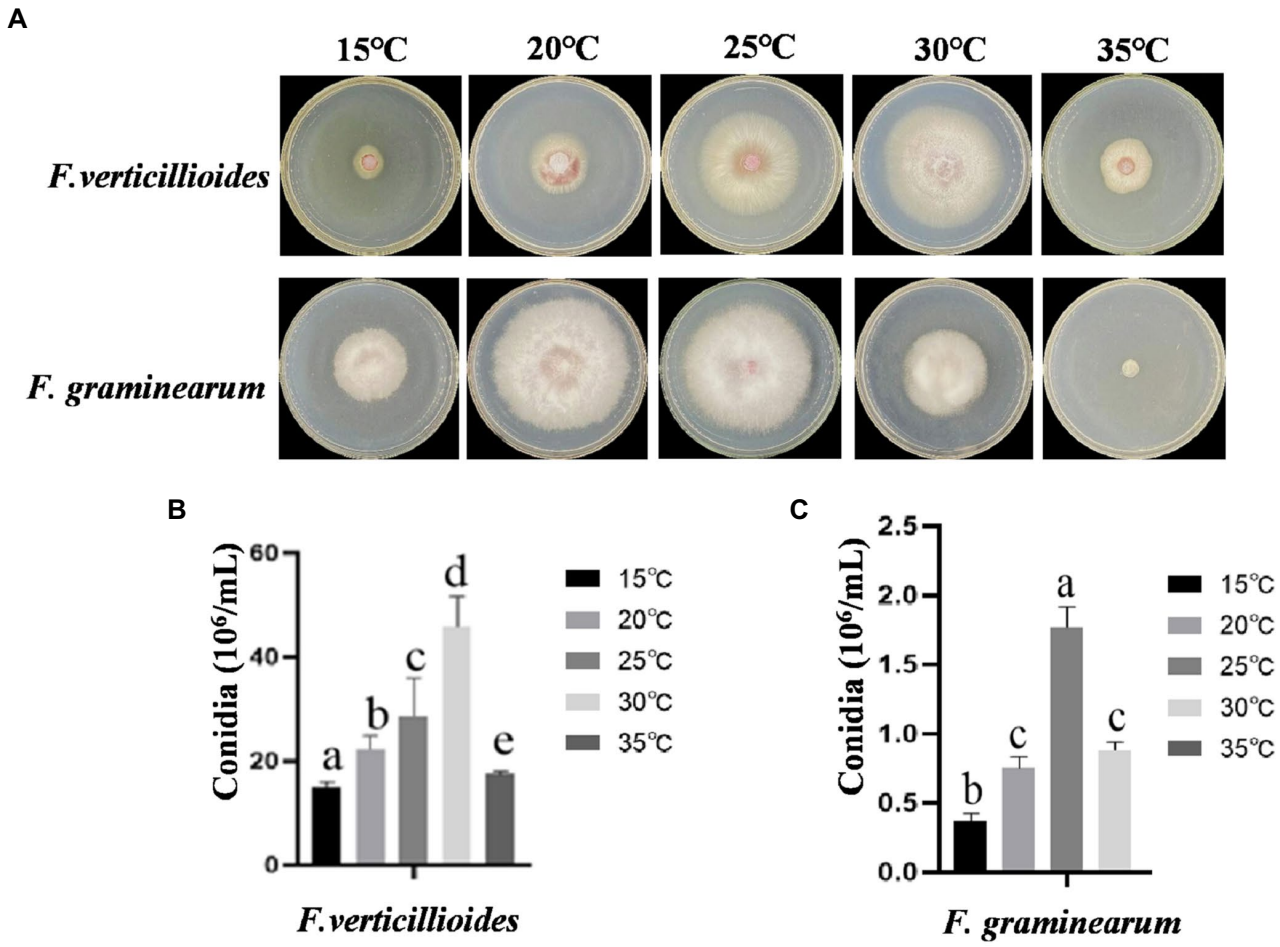
The effect of culturing temperatures on growth and conidial production of *Fusarium verticillioides* and *Fusarium graminearum*. **(A)** Growth of *F. verticillioides* and *F. graminearum* cultivated at 15–30°C. Pictures were taken at 4 days after the initial inoculation. **(B)** Conidial production of *F. verticillioides* grown at 15–30°C. **(C)** Conidial production of *F. graminearum* grown at 15–30°C. Values are means, with standard error bars (*n* = 3). Columns with non-matching letters indicate a significant difference at *p* < 0.05.

The effects of temperature on growth and conidial production of Fv and Fg were evaluated within a range of 15–35°C. The results showed that the mycelia of Fv grew fastest at 30°C, with little growth below 15°C. However, the optimum temperature for Fg growth was 20°C and mycelia showed little growth at 35°C (Figure 1A). The mycelial growth rates of Fv and Fg were fastest at about 4 days at optimum growth temperature, based on the fitting of colony growth data (Supplementary Figure 1). The optimum temperatures for Fv and Fg conidial production were 30 and 25°C, respectively (Figure 1B), with no conidial production by Fg at 35°C. The results show clear differences of temperature on the growth of the two species.

### Effects of temperature on maize stalk rot and ear rot

**Figure 2:**
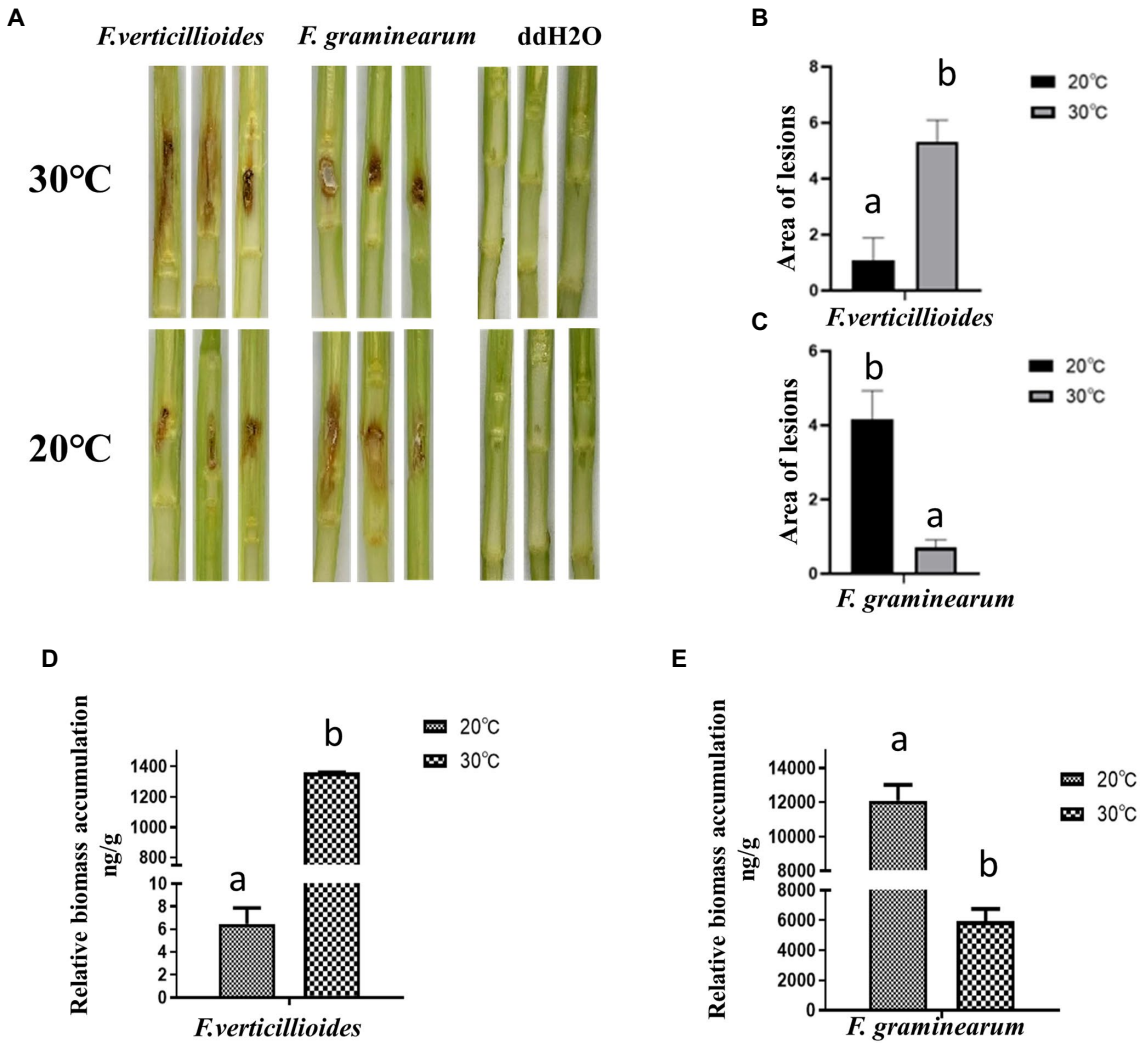
The effect of culturing temperatures on pathogenicity of *F. verticillioides* and *F. graminearum.* **(A)** Representative phenotypes of maize stems inoculated with conidial suspensions of *F. verticillioides* and *F. graminearum.* Maize stems were inoculated with sterile water as control. Photos were taken 4 d after inoculation; The areas of the lesions caused by *F. verticillioides* **(B)** and *F. graminearum* **(C)** were measured. The biomass of maize kernels infected with conidial suspensions of *F. verticillioides* **(D)** *and F. graminearum* **(E)** were measured. Values are means, with standard error bars (n = 3). Columns with non-matching letters indicate a significant difference at *p* < 0.05.

The effect of temperature on pathogenicity of the two *Fusarium* species was evaluated at 20 and 30°C, by inoculating maize stems and kernels. Visual disease symptoms were more obvious for Fv when cultivated at 30°C for 4 days, but for Fg, greater pathogenicity was observed at 20°C (Figure 2A). The warmer temperature treatment (30°C) resulted in significantly larger lesion areas of maize stems inoculated with Fv (Figure 2B). Unlike Fv, the lesion areas caused by Fg were about 75% smaller at the warm temperature treatment compared to those in the cool treatment (Figure 2C). The biomass of Fv in infected maize kernels at 30°C was also significantly greater than at 20°C (Figure 2D), which was the inverse of what was observed for Fg (Figure 2E). These results indicated the optimum temperatures for growth of these two species were the same as the optimum temperatures for pathogenicity, 30°C for Fv and 20°C for Fg.

**Figure 3:**
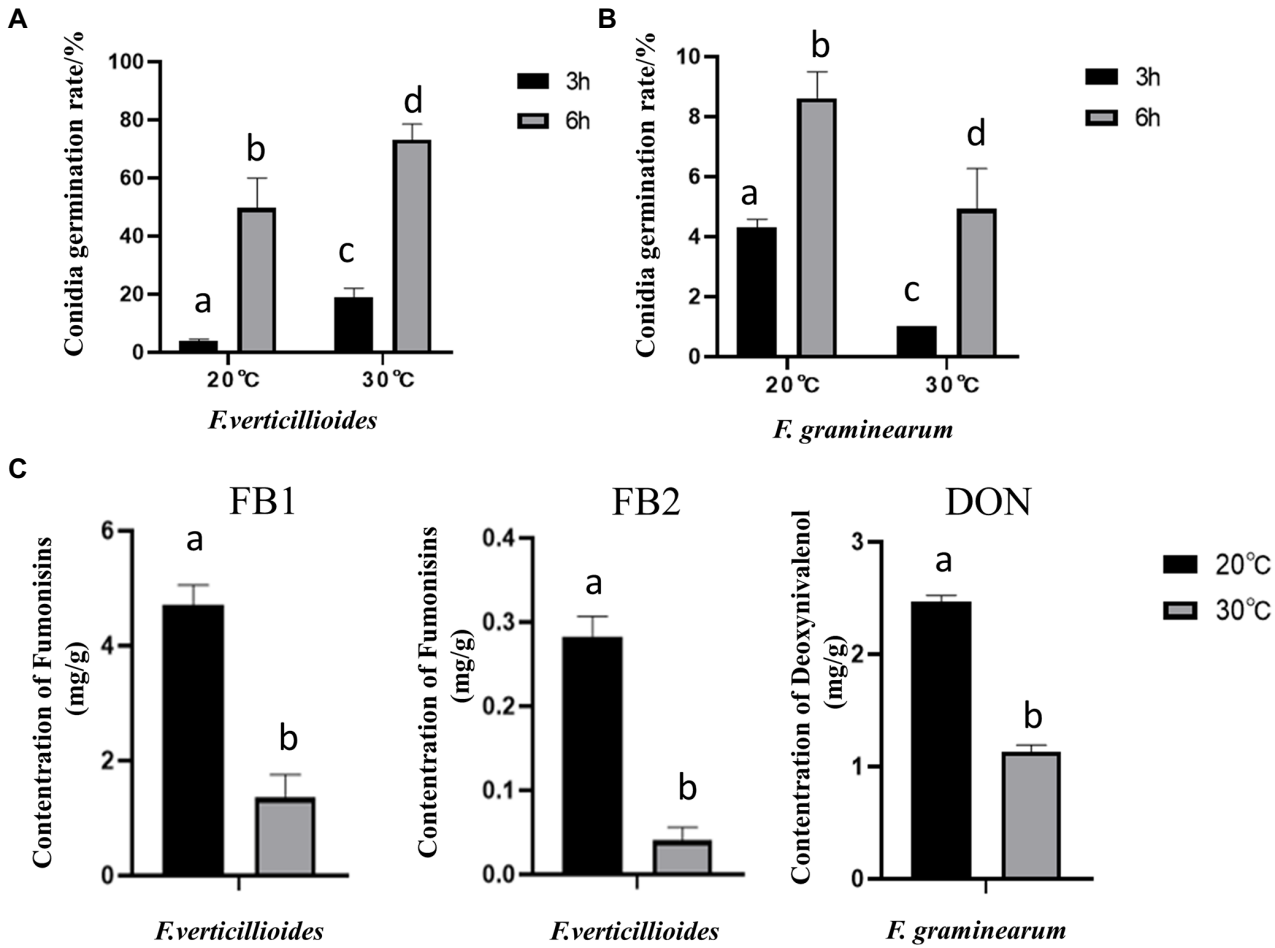
The effect of culturing temperature on conidial germination and mycotoxin production of *F. verticillioides* and *F. graminearum*. Conidial germination of *F. verticillioides* **(A)**, and *F. graminearum* **(B)**, under an oligotrophic condition. The conidia were inoculated on a water agar plate and cultured at 20°C and 30°C to determine the germination percentage; **(C)** the Fumonisins B1 (FB1) and Fumonisins B2 (FB2) content produced by *F. verticillioides* and the Deoxynivalenol (DON) content produced by *F. graminearum* under 20 and 30°C, respectively. Values are means, with standard error bars (*n* = 3). Columns with non-matching letters indicate a significant difference at *p* < 0.05.

Further results showed that compared with at 20°C, the conidial germination rate of Fv was higher at 30°C (Figure 3A), with a rate of 73.24% at 30°C for 6 h. In contrast, the germination rate of Fg was higher at 20°C (Figure 3B). These results are consistent with the differences in pathogenicity at the two different temperatures. Interestingly, both Fv and Fg produced more mycotoxin under the cool temperature treatment (Figure 3C). After incubation at 20°C for 9 days, Fv produced 4.8 mg/g of FB1 and 0.3 mg/g of FB2, and Fg produced 1.18 mg/g of DON, amounts that were all significantly higher than those produced after incubation at 30°C.

### *Fusarium verticillioides* and *Fusarium graminearum* transcriptome analysis

**Figure 4:**
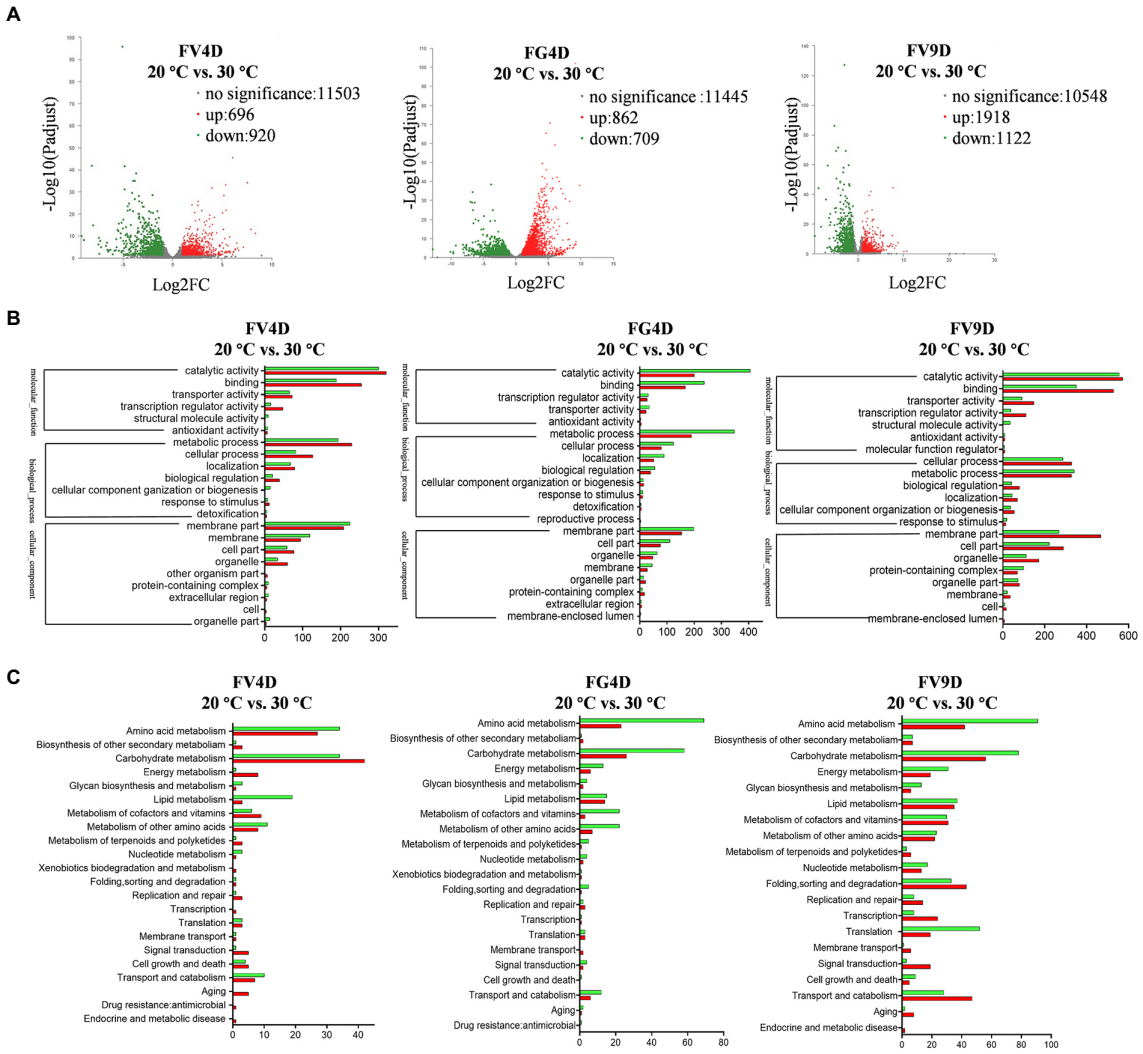
Transcriptome profile of digital RNA-Seq data. **(A)** Volcano plot of digital RNA-seq data. The *X*-axis represents the log2-fold change, and the *Y*-axis represents –log10 significance. Up-regulated genes, down-regulated genes, and genes without significant change are shown as red, green, and grey dots, respectively; **(B)** the GO enrichment analysis of differently expressed genes (DEGs); **(C)** the KEGG enrichment analysis of DEGs. FV4D: DEGs by comparing samples of *F. verticillioides* cultivated for 4 days at 20°C with samples of *F. verticillioides* cultivated for 4 days at 30°C, FG4D: DEGs by comparing samples of *F. graminearum* cultivated for 4 days at 20°C with samples of *F. graminearum* incubated for 4 days at 30°C, FV9D: DEGs by comparing samples of *F. verticillioides* incubated for 9 days at 20°C with samples of *F. verticillioides* incubated for 9 days at 30°C.

To study the differential selection of optimal temperature at the transcriptional level, RNA-Sequencing was used to analyze mycelia of Fv and Fg grown at 20°C and 30°C, respectively, for 4 days, which corresponds to their maximum radial growth rates. We also analyzed samples of Fv cultivated for 9 days at 20 and 30°C, to study the effect of temperature on Fv on a later phase of growth phase. The annotated unigenes found for all samples are listed in Supplementary Table S2. For each species, DEGs were identified by comparing the expression levels of genes grown at 20°C with the expressed levels of genes in the cultures grown for the same amount of time at 30°C (Figure 4A). For the group of FV4D (FV4D-20 vs. FV4D-30), 1,616 genes exhibited differential expression, with 696 genes upregulated and 920 downregulated at 30°C. For the group of Fg4D (FG4D-20 vs. FG4D-30), 1,571 genes were differently expressed, with 862 upregulated and 709 downregulated at 30°C. Fv grown for 9 days at the two different temperatures similarly showed even greater changes. For the group of FV9D (FV9D-20 vs. FV9D-30), there were 3,040 genes differently expressed, with 1918 upregulated and 1,122 downregulated at 30°C.

GO analysis revealed similar term enrichment of DEGs in FV4D, FG4D, and FV9D (Figure 4B). The DEGs associated with cellular component were mainly distributed within the category “membrane part” (433 in FV4D, 353 in FG4D, and 736 in FV9D); DEGs associated with molecular function were mainly enriched in “catalytic activity” (620 in FV4D, 605 in FG4D, and 1,127 in FV9D) and “binding” (444 in FV4D, 405 in FG4D, and 880 in FV9D). DEGs associated with molecular function were mainly enriched in “metabolic process” (424 in FV4D, 538 in FG4D, and 668 in FV9D) and “cellular process” (209 in FV4D, 203 in FG4D, and 615 in FV9D).

KEGG analysis of the identified DEGs was performed next to identify important metabolic pathways, as shown in Figure 4C. For all the groups, the main enriched terms of second category pathways were “carbohydrate metabolism” and “amino acid metabolism.” There were both up-regulated and down-regulated DEGs related to “carbohydrate metabolism” (up-regulated: 42 in FV4D, 26 in FG4D, 56 in FV9D; down-regulated: 34 in FV4D, 58 in FG4D, and 78 in FV9D) and related to “amino acid metabolism” (up-regulated: 27 in FV4D, 23 in FG4D, and 42 in FV9D; down-regulated: 34 in FV4D, 69 in FG4D, and 91 in FV9D). In all groups, of the DEGs related to “amino acid metabolism,” there were more down-regulated DEGs than up-regulated DEGs when grown at 30°C. There were more up-regulated DEGs than down-regulated DEGs in pathways related to “carbohydrate metabolism” and “energy metabolism” for FV4D, but the opposite was seen for FG4D and FV9D.

### Analysis and classification of major metabolism pathways differently affected by temperature in Fv and Fg

Comparison of the top 10 most enriched metabolic pathways for FV4D and FG4D (Table 1) showed the enrichment of up-regulated DEGs in the metabolic pathways of “tyrosine metabolism” and “glycolysis/gluconeogenesis,” and enrichment of down-regulated DEGs in the metabolic pathways of “glycine, serine, and threonine metabolism,” “arginine and proline metabolism,” “beta-alanine metabolism” and” tryptophan metabolism.” The DEGs in these metabolic pathways tended to change in the same way in response to temperature despite the different optimum temperatures of Fv and Fg of 30 and 20°C, respectively.

**Table 1:** The top 10 most enriched metabolism pathways of DEGs analyzed by Kyoto Encyclopedia of Genes and Genomes (KEGG).

FV4D				FG4D				FV9D			
Up-regulated		Down-regulated		Up-regulated		Down-regulated		Up-regulated		Down-regulated	
Pathway	DEGs number	Pathway	DEGs number	Pathway	DEGs number	Pathway	DEGs number	Pathway	DEGs number	Pathway	DEGs number
Amino sugar and nucleotide sugar metabolism	9	Arginine and proline metabolism	13	Tyrosine metabolism	10	Glyoxylate and dicarboxylate metabolism	17	Protein processing in endoplasmic reticulum	26	Ribosome	30
Starch and sucrose metabolism	7	Tyrosine metabolism	12	Amino sugar and nucleotide sugar metabolism	10	Glycine, serine and threonine metabolism	15	Autophagy, yeast	18	Glycolysis/Gluconeogenesis	18
Pentose phosphate pathway	7	Phenylalanine metabolism	12	Tryptophan metabolism	9	Arginine and proline metabolism	15	MAPK signaling pathway, yeast	15	Arginine and proline metabolism	17
Glycolysis / Gluconeogenesis	7	Tryptophan metabolism	11	Glycine, serine and threonine metabolism	5	Tryptophan metabolism	12	Amino sugar and nucleotide sugar metabolism	14	Cysteine and methionine metabolism	16
Alanine, aspartate and glutamate metabolism	6	Amino sugar and nucleotide sugar metabolism	9	Fatty acid degradation	5	Pyruvate metabolism	12	Peroxisome	14	Glycine, serine and threonine metabolism	15
Glyoxylate and dicarboxylate metabolism	6	beta-Alanine metabolism	8	Phenylalanine metabolism	5	Valine, leucine and isoleucine degradation	11	Glycerophospholipid metabolism	14	Tyrosine metabolism	14
Tyrosine metabolism	5	Fatty acid degradation	7	Arginine and proline metabolism	5	Glutathione metabolism	11	Spliceosome	13	Histidine metabolism	14
MAPK signaling pathway, yeast	5	Glycine, serine and threonine metabolism	6	Glycolysis / Gluconeogenesis	5	Cysteine and methionine metabolism	10	Purine metabolism	12	Starch and sucrose metabolism	14
Peroxisome	5	Pentose and glucuronate interconversions	5	Lysine degradation	4	Valine, leucine and isoleucine biosynthesis	10	RNA transport	11	Lysine degradation	13
Longevity regulating pathway—multiple	5	Galactose metabolism	5	Glycerolipid metabolism	4	beta-Alanine metabolism	9	Ubiquitin mediated proteolysis	11	Galactose metabolism	13

FV4D: DEGs by comparing samples of *Fusarium verticillioides* cultivated for 4 days at 20°C with samples of *F. verticillioides* cultivated for 4 days at 30°C, FG4D: DEGs by comparing samples of *Fusarium graminearum* cultivated for 4 days at 20°C with samples of *F. graminearum* cultivated for 4 days at 30°C, FV9D: DEGs by comparing samples of *F. verticillioides* cultivated for 9 days at 20°C with samples of *F. verticillioides* cultivated for 9 days at 30°C.

Comparison of the top 10 most enriched metabolic pathways for FV4D and FV9D (Table 1) showed the enrichment of up-regulated DEGs in the metabolic pathways of “amino sugar and nucleotide sugar metabolism,” “MAPK signaling pathway-yeast,” “peroxisome,” and enrichment of down-regulated DEGs in the metabolic pathways for “arginine and proline metabolism,” tyrosine metabolism,” “glycine, serine, and threonine metabolism” and “galactose metabolism.” Compared to FV4D, there were more DEGs down-regulated in FV9D in the pathways of “glycolysis/gluconeogenesis,” “tyrosine metabolism,” and “starch and sucrose metabolism.” Because FV produced higher levels of fumonisins at 20°C than at 30°C, we focused on genes involved in toxin synthesis. The fumonisin biosynthetic gene cluster includes 17 genes, of which FVEG_00321 (FUM10), FVEG_00324 (FUM13), FVEG_00328 (FUM18) and FVEG_00329 (FUM19) were expressed at lower levels when Fv was grown at 30°C at 4 and 9d of growth compared to the levels when Fv was grown at 30°C. These changes in gene expression were consistent with higher toxin production for Fv cultivated at lower temperature.

**Figure 5:**
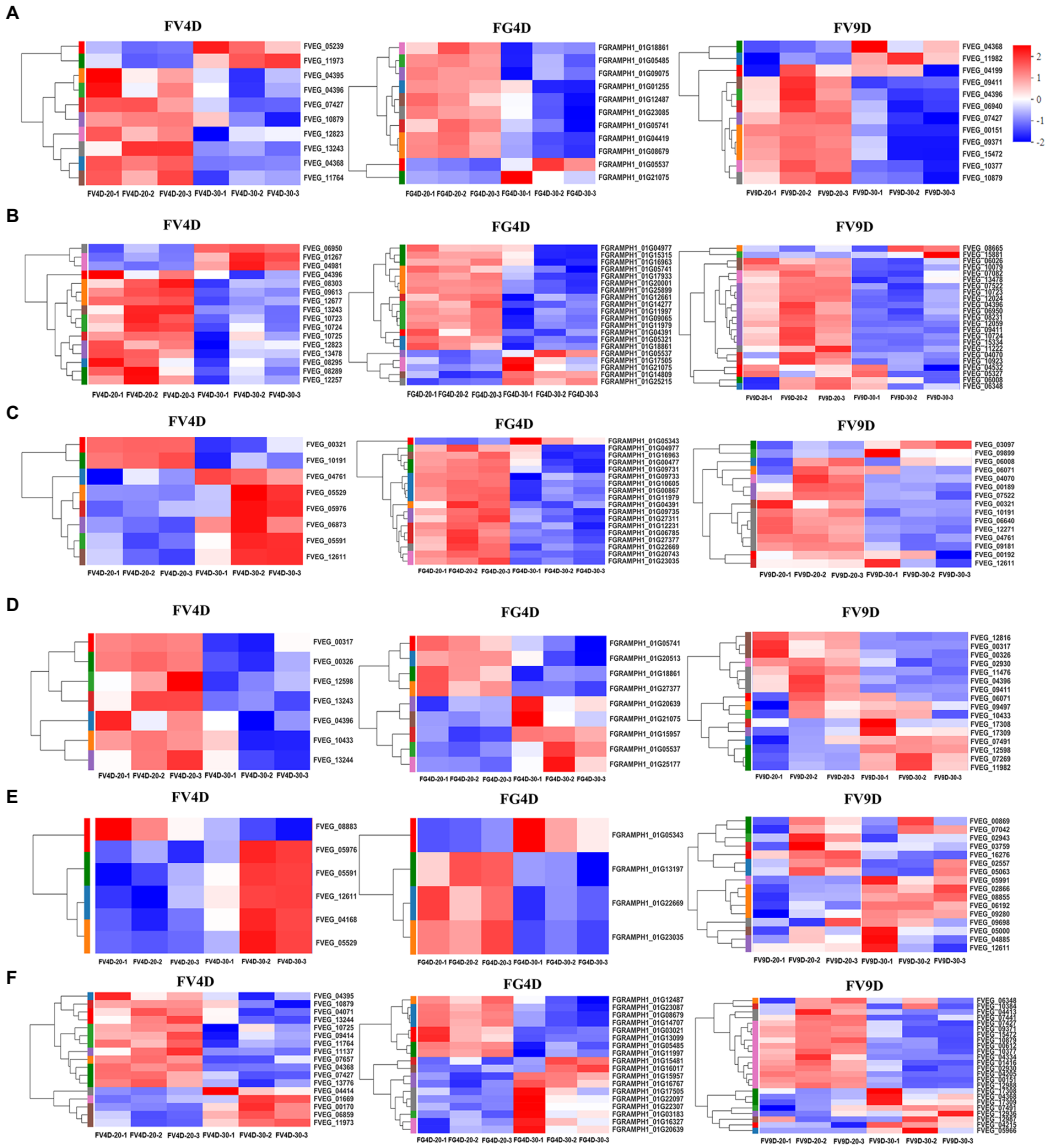
Heat map of differently expressed genes enriched in beta-alanine metabolism **(A)**, Arginine and proline metabolism **(B)**, Glyoxylate and dicarboxylate metabolism **(C)**, Fatty acid degradation **(D)**, MAPK signaling pathway–yeast **(E)**, Tyrosine metabolism **(F)**, pathways. Red to blue indicates expression level from high to low. FV4D-20: *F. verticillioides* cultivated for 4 days at 20°C, FV4D-30: *F. verticillioides* cultivated for 4 days at 30°C, FG4D-20: *F. graminearum* incubated for 4 days at 20°C, FG4D-30: *F. graminearum* cultivated for 4 days at 30°C, FV9D-20: *F. verticillioides* cultivated for 9 days at 20°C, FV9D-30: *F. verticillioides* cultivated for 9 days at 30°C. FV4D: DEGs by comparing samples FV4D-20 with samples of FV4D-30. FG4D: DEGs by comparing samples FG4D-20 with samples of FG4D-30. FV9D: DEGs by comparing samples FV9D-20 with samples of FV9D-30.

**Figure 6:**
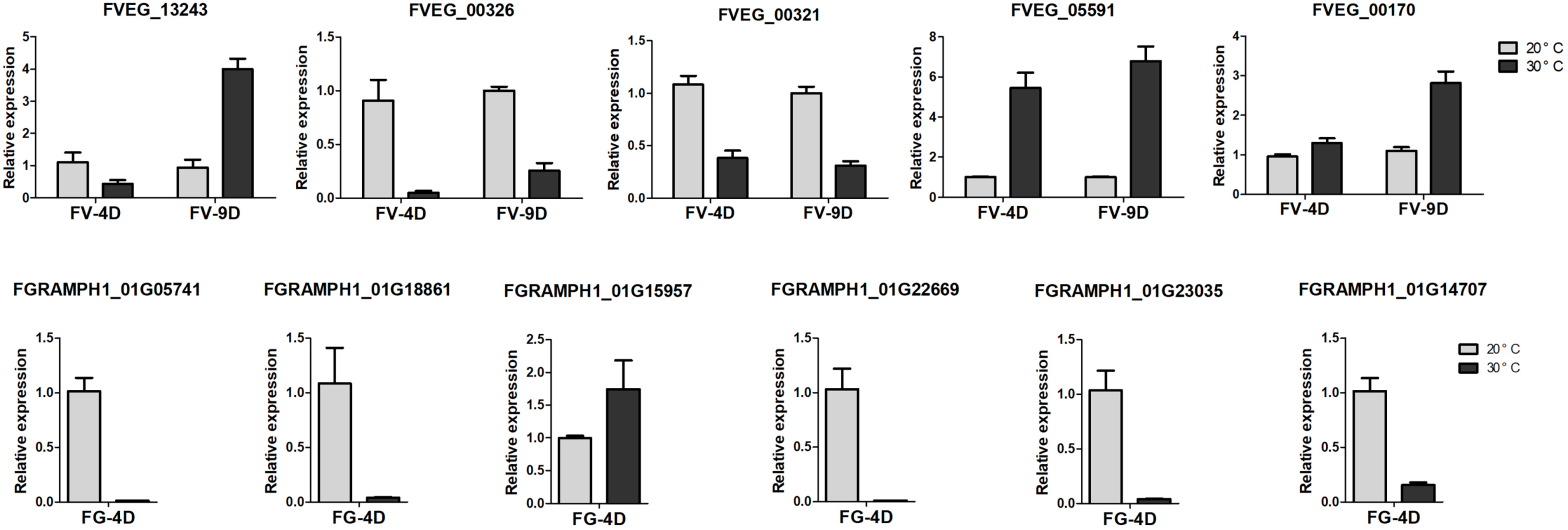
The relative expression levels of differently expressed genes detected by qRT-PCR. The data were obtained from three biological replicates and Actin of F. verticillioides and GAPDH of F. graminearum were used as the internal reference. FV-4D: F. verticillioides cultivated for four days, FV-9D: F. verticillioides cultivated for nine days. FG-4D: F. graminearum cultivated for four days.

There was enrichment of DEGs in different pathways affected by temperature (Figure 5) and the relative expression levels of some of them was validated by qRT-PCR (Figure 6). DEGs were mainly down-regulated in the “beta-alanine metabolism” pathway (map00410; Figure 5A) and “arginine and proline metabolism” pathway (map00330; Figure 5B). The expression levels of FGRAMPH1_01G14277, FGRAMPH1_01G12661, and FGRAMPH1_01G09065, all encoding amidase, were reduced at 30°C in FG4D. The homologous genes in FV4D (FVEG_08295, FVEG_08289, FVEG_12257, FVEG_12677, and FVEG_13478) and in FV9D (FVEG_15334, FVEG_10923, and FVEG_13478) were also down-regulated at 30°C relative to the levels at 30°C. Genes encoding aldehyde dehydrogenase (NAD+) exhibited similar expression patterns, including FVEG_09411, FVEG_04396, FVEG_13243 and FGRAMPH1_01G05741, and FGRAMPH1_01G18861.

There were also temperature-responsive differences between FV4D and FG4D, such as DEGs related to the pathways of “glyoxylate and dicarboxylate metabolism” (map00630; Figure 5C) and “fatty acid degradation” (map00071; Figure 5D). All DEGs of FV4D in “fatty acid degradation” pathway were down-regulated at 30°C, while many DEGs of FG4D in this pathway were up-regulated. The down-regulated DEGs included genes encoding aldehyde dehydrogenase(such as FVEG_04396, FGRAMPH1_01G21075 and FGRAMPH1_01G05741), long-chain fatty acid CoA synthetase (FVEG_00326) and long-chain fatty acid acyl-CoA synthetase family protein (FVEG_00321). The up-regulated DEGs in FG4D included genes encoding cinnamyl alcohol dehydrogenases (FGRAMPH1_01G20639 and FGRAMPH1_01G05537), and zinc-binding dehydrogenase (FGRAMPH1_01G15957), which all belonged to the medium-chain fatty acid dehydrogenase/reductase family.

In addition, 4 genes related to “MAPK signaling pathway–yeast” pathway (map04011; Figure 5E), FVEG_05591, FVEG_12611, FVEG_05976, and FVEG_05529, which encode catalase, were up-regulated both in FV4D and FV9D but their homologous genes FGRAMPH1_01G22669 and FGRAMPH1_01G23035 exhibited the opposite tend of expression. Since the optimum temperatures of *F. verticillioides* and *F. graminearum* were 30 and 20°C respectively, the observed differences in expression in genes related to these metabolism pathway may partially explain the differences in growth at different temperatures.

Additional DEGs exhibited both similar and opposite expression patterns in the two species. For example the expression patterns of several genes related to “tyrosine metabolism” (map00350; Figure 5F) showed opposite trends in the two species, such as genes encoding succinate/glutarate-semialdehyde dehydrogenase (FVEG_01669 and FVEG_00170 in Fv, and FGRAMPH1_01G23087 and FGRAMPH1_01G14707 in Fg), genes encoding homogentisate 1,2-dioxygenase (FVEG_09414 and FGRAMPH1_01G22307), and genes encoding alcohol dehydrogenase (propanol-preferring; FVEG_13244, FGRAMPH1_01G20639, and FGRAMPH1_01G15957). However, the expression of homologous genes that encode primary-amine oxidase were reduced in both species at 30°C (FVEG_11764, FVEG_04368, FVEG_10879, and FVEG_04395 in FV4D, FVEG_15472, FVEG_10879, and FVEG_09371 in FV9D, and FGRAMPH1_01G12487 and FGRAMPH1_01G08679 in FG4D).

## Discussion

The selectivity of optimum temperature of fungi, even within the same genus, has long been reported. The optimum temperature for growth of *F. pseudograminearum* is 15°C (Sabburg et al., 2015); *F. oxysporum* exhibits highest growth rate at 25°C and strongest pathogenicity at 30°C (Jimenez et al., 2019); the optimum temperature is 30°C for growth of *F. proliferatum* and *F. verticillioides* (Samapundo et al., 2005). In our study, the optimum temperatures for growth, spore germination, and pathogenicity were 30°C for Fv and 20°C for Fg. Environmental temperature has an important effect on biodistribution, especially the geographic distribution of animals and plants. The different optimum temperatures of *Fusarium* species suggest that temperature may significantly affect the distribution of pathogens that cause maize rot in different regions and years. In Eastern European countries with a temperate continental climate, such as Russia and Ukraine *F. graminearum* is a major pathogen of maize stem rot. In Western European countries with a temperate monsoon climate, *F. verticillioides* is the major stem rot pathogen (Kommedahl et al., 1978). In China, in the Huang-Huai-Hai region located at 114°E-120°E and 34°N-40°N, the average temperature in August is 23–32°C, and *F. verticillioides* is the main pathogen causing maize stem rot (Liu et al., 2019). The maize belt in northeast China is located at 118°E-135°E and 48°N-55°N with an average temperature of 19–27°C in August, and *F. graminearum* is the main pathogen causing maize stem rot (Liu et al., 2014). However, interspecific differences in how performance metrics respond to temperature do not always align with the distribution of pathogens, suggesting other ecological factors can also act as important drivers (Bååth, 2018). The pathogenicity of fungi is related to temperature as well as ecological factors such as humidity (water potential) and the host plant and the soil microbial community, which may interact with temperature to ultimately determine distribution. A larger sample of fixed pathogenicity testing for conditions may reveal the basis for differences.

Although transcriptome sequencing has been widely used in the study of *Fusarium* disease, this is the first attempt to use association analysis of transcriptome data to explain the optimum temperature selection of two similar *Fusarium* species (Fv and Fg) under test conditions that exclude differences due to light and water potential. The results of GO analysis showed that DEGs were enriched in categories of “membrane part,” “catalytic activity,” and “metabolic process” in the three groups. For DEGs related to the category of “membrane part,” there were more down-regulated DEGs than up-regulated DEGs when the two species were grown at 30°C for 4 days, compared with 20°C. Temperature changes are closely related to plasma membrane fluidity, and plasma membrane fluidity can be used as an indicator of fitness for survival in extreme environments (Turk et al., 2011). Membrane fluidity is also involved in the temperature sensing of *Candida albicans* (Leach and Cowen, 2014). Our identification of DEGs enriched in membrane components suggests that growth at different temperatures can affect the plasma membranes of both Fv and Fg. The influence of cell membrane fluidity on the growth of Fv and Fg at different cultivated temperatures was investigated, using DMSO to inhibit cell membrane fluidity and BA to increase cell membrane fluidity (Supplementary Figure 3). It was found that when the membrane fluidity decreased (treated with DOMS), the growth of Fv and Fg showed similar inhibition rate at different cultivated temperatures. But after the membrane fluidity increased (treated with BA), the growth of Fv cultivated at higher than its own optimal temperature was not inhibited, while Fg was significantly inhibited and basically stopped growing. Fatty acids have been reported to regulate the membrane fluidity and play important roles in response to changes in environmental temperature (Mejía-Barajas et al., 2018). Cells alter fatty acid composition to maintain the cell membrane fluidity (Torija et al., 2003). In our study, the DEGs were also enriched in “fatty acid degradation” pathway. Some DEGs encoding enzymes that catalyze long-chain fatty acids was down-regulated, while some DEGs encoding enzymes that catalyze medium-chain fatty acids was up-regulated. These DEGs appear to be driving the temperature disparities, which needs further study.

Kyoto Encyclopedia of Genes and Genomes analysis also showed enrichment of DEGs in carbohydrate and amino acid metabolism with growth at different temperatures. Amino acid metabolism can also respond to temperature. Transcriptome analysis shows that increasing growth temperature alters amino acid metabolism in *Aspergillus flavus* to maintain intracellular osmotic balance (Bai et al., 2015). Here, DEGs were mainly down-regulated in both Fv and Fg in amino acid metabolism pathways of “beta-alanine metabolism” and “arginine and proline metabolism” when grown at warm temperature. Some homologous genes in Fv and Fg were similarly regulated, such as the genes encoding aldehyde dehydrogenase (NAD^+^) down-regulated at 30°C in all groups. Aldehyde dehydrogenase oxidizes aldehydes to the corresponding carboxylic acids and is involved in resistance against aldehydes derived from lipid peroxidation and ROS, thus playing a crucial role in response to low temperature stress in potato and tobacco (Muzio et al., 2012; Guo et al., 2020). Further studies are needed to determine why these genes show the same pattern in response to temperature despite different growth rates at different growth temperatures.

Some DEGs exhibited opposite patterns in Fv and Fg, including genes in carbohydrate metabolism, such as “glyoxylate and dicarboxylate metabolism.” DEGs with opposite expression patterns in Fv and Fg may be regulated by temperature and related to growth. The expression levels of FVEG_01669 and FVEG_00170, encoding succinate-semialdehyde dehydrogenases (SSADHs) in *F. verticillioides*, were higher at 30°C, but expression levels of homologous genes in Fg (FGRAMPH1_01G23087 and FGRAMPH1_01G14707) were higher at 20°C. SSADHs play a crucial role in the functioning of the GABA pathway *in vivo* (Wang et al., 2015). SSADH Sdh1 in *Stagonospora nodorum* is involved in responding to environmental stress, and species with Sdh1 mutants are more susceptible to reactive oxygen species stress, but less affected by increased growth temperature (Mead et al., 2013). This suggests that the homologous DEGs with opposite expression patterns represent genes regulated by temperature.

In our study, the expression levels of DEGs in “MAPK signaling pathway–yeast” pathway were mainly increased when Fv and Fg were grown at their optimum temperatures, and more DEGs were found when Fv was cultivated for 9 days. The MAPK signaling pathway in eukaryotic organisms is one of the most important and evolutionarily conserved mechanisms to sense extracellular information and activate different transcription factors to regulate gene expression; this pathway is closely related to the pathogenicity of plant pathogenic fungi (Martínez-Soto and Ruiz-Herrera, 2017; Lemos et al., 2018). The influence of MAPK signaling pathway on the growth of Fv and Fg at different cultivated temperatures was investigated using the inhibitor U0126 (Supplementary Figure 4). It was found Fv and Fg were inhibited by 3 μMU0126 at 20°C and 30°C, and under 30°C, the inhibition rates of both Fv and Fg increased than that of 20°C. But when treated with 1 μMU0126, Fv was inhibited at 20°C and 30°C, while Fg grew faster than control under 30°C when treated with 1 μM U0126. As the differently expression level of genes in MAPK signaling pathway analyzed by transcriptome suggested that MAPK signaling pathway may be a major temperature signal pathway to regulate genes involved in growth, further study on the difference of genes regulated by MAPK between the two species will provide a more definite explanation.

Many studies have focused on temperature stress, but few studies have investigated different thermal niches among different organisms and their regulatory mechanisms. Prokaryotes growing at high temperatures typically have higher GC content, as GC pairs are more thermally stable than AT pairs in DNA double helix structures (Hu et al., 2022). According to studies on *Droseraceae*, genomic GC content is positively correlated with genome size and annual temperature fluctuations, with GC content ranging between 37.1 and 44.7% (Veleba et al., 2017). However, the genomes of Fv and Fg are 48.6857 and 48.3278% GC, and *F. verticillioides* and *F. graminearum* orthologues share 85% nucleotide sequence identity (Ma et al., 2010). Thus, variation in GC content may explain extreme cases of temperature adaptation, but other factors may be more important for more similar organisms. The goal of this work was to explore the mechanism of different temperature selectivity of related species at the transcriptional level. Most stressor response-related genes that were previously reported to respond to temperature change did not exhibit significant differences in expression levels in this study. For example, heat shock proteins (HSP) are involved in the folding and unfolding of proteins, and play an integral role in the response to temperature and other stressors (Mota et al., 2019). However, in our study, HSP genes did not change significantly (Supplementary Figure 5). Compared to HSP, the overall temperature-dependent differences in amino acid and carbohydrate metabolism may represent another strategy to explain differences in thermal niches. This transcriptome analysis of Fv and Fg provides data and suggests potential molecular mechanisms to explain differences in ecological success among closely related and ecologically similar species.

In general, the differences of growth and pathogenicity between Fv and Fg affected by temperature were investigated and analyzed by RNA-seq. The optimum temperature of growth and disease occurrence in maize stem and kernels was 30°C for Fv, and 20°C for Fg, but both species both produced more toxins at 20°C. Transcriptome analysis showed that the different temperature caused DEGs to be mainly enriched in membrane components, catalytic activities, and metabolic processes in the two species, and there were more down-regulated DEGs enriched in membrane under warmer conditions. KEGG analysis of Fv and Fg showed that temperature affects carbohydrate and amino acid metabolism of these two fungal species in different ways. In both Fv and Fg, the expression of many DEGs enriched in amino acid metabolism decreased when grown at the warmer temperature, including genes related to beta-alanine metabolism and arginine and proline metabolism. The pattern of DEGs in glyoxylate and dicarboxylate metabolism and fatty acid degradation was related to the growth state. Differences in genes related to these pathways may explain the distinct thermal ecological niches of these two closely related fungal species.

## Data availability statement

The datasets presented in this study can be found in online repositories. The names of the repository/repositories and accession number(s) can be found at: https://www.ncbi.nlm.nih.gov/, PRJNA872283, PRJNA873264 and PRJNA873191.

## Author contributions

NL, ZC, and JD conceived and designed the study and wrote the paper. YC and JL performed the main experiments. QS, MS, BZ, and HJ performed some experiments and involved in data analyses. All authors contributed to the article and approved the submitted version.

## Funding

This work was supported by grants from the China Agriculture Research System (CARS-02), Key Research and Development Projects of Hebei (20326502D), “Three Talents Project” funded project of Hebei Province (A202102005), The Independent Research Projectin State Key Laboratory of North China Improvement and Regulation (No. NCCIR2020ZZ-12), and The Natural Science Foundation of Hebei Province (No. C2021204093).

## Conflict of interest

The authors declare that the research was conducted in the absence of any commercial or financial relationships that could be construed as a potential conflict of interest.

## Publisher’s note

All claims expressed in this article are solely those of the authors and do not necessarily represent those of their affiliated organizations, or those of the publisher, the editors and the reviewers. Any product that may be evaluated in this article, or claim that may be made by its manufacturer, is not guaranteed or endorsed by the publisher.
