# Supplementary material for: Transcriptional differences between major Fusarium pathogens of maize, Fusarium verticillioides and Fusarium graminearum with different optimum growth temperatures
Source: Front Microbiol. 2022 Dec 1;13:1030523. doi: 10.3389/fmicb.2022.1030523 (PMC9751597; doi:10.3389/fmicb.2022.1030523)
Supplement: Supplementary file 1 [file Data_Sheet_1.docx]

Supplementary Material

## Supplementary Figures


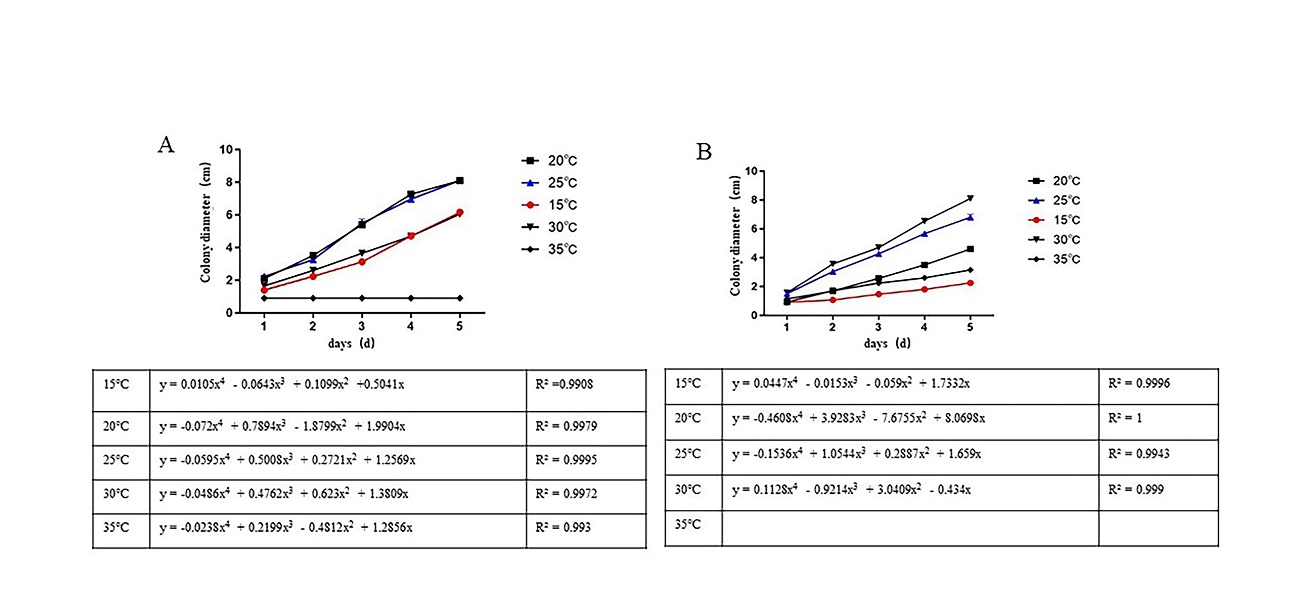
**Supplementary Figure 1.** Effects of different temperatures on radial mycelial growth of *F.verticillioides* (A) and *F. graminearum* (B) and the fitting equations of growth curves.
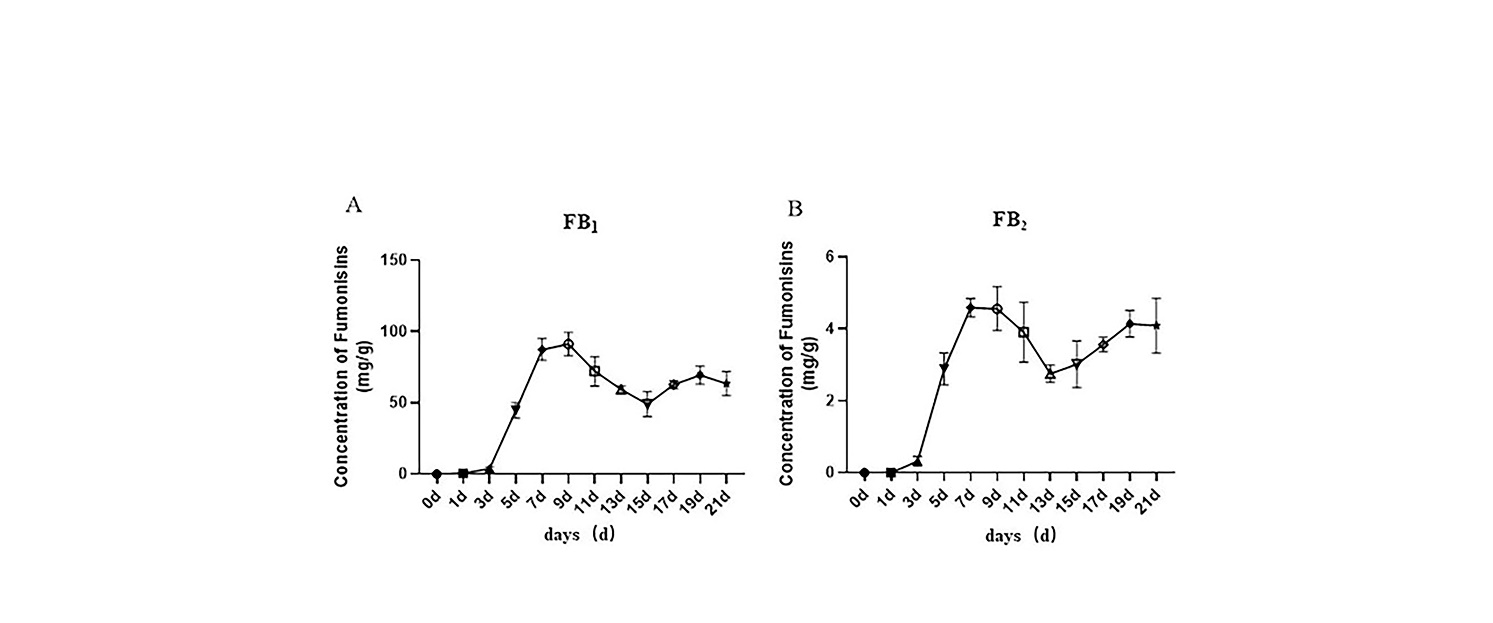
**Supplementary Figure 2.** The levels of Fumonisin B1 (A) and Fumonisin B2 (B) produced by F. verticillioides cultured for different lengths of time at 20 °C.

**Supplementary Figure 3.** The influence of cell membrane fluidity on the growth of *F. verticillioides* and *F. graminearum* at different incubated temperatures. 600 mM dimethylsulfoxide(DMSO) was added in PDA medium for inhibiting cell membrane fluidity, 10 mM benzyl alcohol(BA) for increasing cell membrane fluidity. *F. verticillioides* (A) and *F. graminearum* (B) were incubated at 20 °C and 30 °C to measure the diameters of colonies. And the inhibition rates of DMSO and BA to growth (C) were calculated at 4 days incubated at 20 °C and 30 °C. *F. verticillioides* and *F. graminearum* incubated in PDA medium at the corresponding temperature were used as control.

**Supplementary Figure 4.** The influence of MAKP signaling pathway on the growth of *F. verticillioides* and *F. graminearum* at different incubated temperatures. 1 μM or 3 μM MAKP signaling pathway inhibitor U0126 was added in PDA. *F. verticillioides* and *F. graminearum* were incubated at 20 °C and 30 °C to measure the diameters of colonies, and the inhibition rates of U0126 to growth were calculated at 4 days. *F. verticillioides* and *F. graminearum* incubated in PDA medium at the corresponding temperature were used as control.

**Supplementary Figure 5.** Heat map of expression levels of differently expressed genes in the family of Heat shock protein 70.

Note: From red to blue, the expression level was from high to low. FV4D-20: F. verticillioides incubated for four days at 20 °C, FV4D-30: F. verticillioides incubated for four days at 30 °C, FG4D-20: F. graminearum incubated for four days at 20 °C, FG4D-30: F. graminearum incubated for four days at 30 °C, FV9D-20: F. verticillioides incubated for nine days at 20 °C, FV9D-30: F. verticillioides incubated for nine days at 30 °C. FV4D: DEGs by comparing samples FV4D-20 with samples of FV4D-30, FG4D: DEGs by comparing samples FG4D-20 with samples of FG4D-30, FV9D: DEGs by comparing samples FV9D-20 with samples of FV9D-30.

**Supplementary Table S1.** Sequences of primers used in this study.

| Genes | Primers sequences(5'to3') |
| --- | --- |
| *Fusarium verticillioides* 7600 | |
| FVEG_00326-F | TGTATATCGGCGGCACACTC |
| FVEG_00326-R | TCATCGCAGTCCAGAACACC |
| FVEG_00321-F | TGGTTGCTGTGATGCCTTCT |
| FVEG_00321-R | TCGCTCTGGTGGTAGGATCA |
| FVEG_13243-F | CGAACGACAACCCCTGAAGA |
| FVEG_13243-R | TGGTGAAGTTGAAGCGCTCT |
| FVEG_05591-F | ATCCCGGAAAGAAGGCCAAG |
| FVEG_05591-R | ACGAAAGGAAGGGCACTCTG |
| FVEG_00170-F | ACAAAGTCATCGGCTCAGCA |
| FVEG_00170-R | ATGCGGGACAACATCACCAT |
| FV-Actin-F | TGCTCCTGAGGCTCTCTTCCA |
| FV-Actin-R | AAGCAAGAATAGAACCACC |
| *Fusarium graminearum* PH-1 | |
| FGRAMPH1_01G15957-F | GCCCTTCAGATCGTTCGTCA |
| FGRAMPH1_01G15957-R | CCTGGTGCCGATCTTCATGT |
| FGRAMPH1_01G05741-F | TCAATCACGGTCCTCAAGCC |
| FGRAMPH1_01G05741-R | CGATGGCCTCCTCTTCTGTC |
| FGRAMPH1_01G18861-F | CCAGGCAGATGTCTACGACC |
| FGRAMPH1_01G18861-R | CTCTTCGCGCATGATGGTTG |
| FGRAMPH1_01G22669-F | TTTCATCGCAAAGCATGGCC |
| FGRAMPH1_01G22669-R | GCGGAACTCAGATCTAGGCC |
| FGRAMPH1_01G23035-F | ACCAAGTTCGTCAAGTGGCA |
| FGRAMPH1_01G23035-R | ACGGGGTTTGTGTCCAACTT |
| FGRAMPH1_01G14707-F | CTTGCTGCTGGTACCCTGAA |
| FGRAMPH1_01G14707-R | GAGCTTGGCAGTGAACTCCT |
| FG-GAPDH-F | GTCCACTCCTACACTGCCAC |
| FG-GAPDH-R | GCCTCCTTGATGACCTGCTT |

**Supplementary Table S2.** Summary statistics of annotation of all unigenes.

| **Number of unigenes annotated by the indicated database** | | | | | | | | | |
| --- | --- | --- | --- | --- | --- | --- | --- | --- | --- |
| species | Training time | Values | Total | GO | COG | KEGG | Nr | Pfam | Swissprot |
| *Fusarium verticillioides* 7600 | 4d | Number | 13119 | 9571 | 12539 | 4440 | 13089 | 9359 | 8119 |
|  |  | Percentage | 100 | 72.96 | 95.58 | 33.84 | 99.77 | 71.34 | 61.89 |
| *Fusarium verticillioides* 7600 | 9d | Number | 13588 | 9323 | 12728 | 4567 | 13588 | 9098 | 8671 |
|  |  | Percentage | 100 | 68.61 | 93.67 | 33.61 | 98.54 | 69.9 | 63.81 |
| *Fusarium graminearum* PH-1 | 4d | Number | 13016 | 8053 | 11590 | 4320 | 12710 | 9098 | 7587 |
|  |  | Percentage | 100 | 61.87 | 89.04 | 33.19 | 97.65 | 69.9 | 58.29 |
